# High-Performance Deep Ultraviolet Photodetector Based on NiO/β-Ga_2_O_3_ Heterojunction

**DOI:** 10.1186/s11671-020-3271-9

**Published:** 2020-02-22

**Authors:** Menghan Jia, Fang Wang, Libin Tang, Jinzhong Xiang, Kar Seng Teng, Shu Ping Lau

**Affiliations:** 1 School of Materials Science and Engineering, Yunnan University, Kunming, 650091 China; 2 Kunming Institute of Physics, Kunming, 650223 China; 3 Yunnan Key Laboratory of Advanced Photoelectric Materials & Devices, Kunming, 650223 China; 4 School of Physics and Astronomy, Yunnan University, Kunming, 650091 China; 5 College of Engineering, Swansea University, Bay Campus, Fabian Way, Swansea, SA1 8EN UK; 6 Department of Applied Physics, The Hong Kong Polytechnic University, Hung Hom, Kowloon, Hong Kong, China

**Keywords:** β-Ga_2_O_3_, NiO, Heterojunction, UV photodetector

## Abstract

Ultraviolet (UV) photodetector has attracted extensive interests due to its wide-ranging applications from defense technology to optical communications. The use of wide bandgap metal oxide semiconductor materials is of great interest in the development of UV photodetector due to their unique electronic and optical properties. In this work, deep UV photodetector based on NiO/β-Ga_2_O_3_ heterojunction was developed and investigated. The β-Ga_2_O_3_ layer was prepared by magnetron sputtering and exhibited selective orientation along the family of ($\overline{2}$ 01) crystal plane after annealing. The photodetector demonstrated good performance with a high responsivity (*R*) of 27.43 AW^−1^ under a 245-nm illumination (27 μWcm^−2^) and the maximum detectivity (D*) of 3.14 × 10^12^ cmHz^1/2^ W^−1^, which was attributed to the p-NiO/n-β-Ga_2_O_3_ heterojunction.

## Background

There have been much research interests in the development of ultraviolet (UV) photodetectors due to their wide-ranging applications, such as missile warning, biochemical analysis, flame and ozone detections, and optical communications. As compared to SiC and GaN semiconductors, UV photodetectors based on wide bandgap metal oxide semiconductors offer many advantages. For example, the metal oxide-based photodetectors do not oxidize easily and exhibit sensitive response. Furthermore, they are easy to operate and can be made small in size [1, 2]. Hence, wide bandgap metal oxides and their devices have attracted much research attention in recent years. To date, metal oxides such as ZnO [3–5], TiO_2_ [6, 7], SnO_2_ [8], NiO [9], and Ga_2_O_3_ [10, 11] have been studied for use as high-performance UV photodetectors. Among them, the stable phase of Ga_2_O_3_ (β-Ga_2_O_3_) is becoming a preferred material for UV photodetector as it is a direct bandgap semiconductor with ultra-wide bandgap of ~ 4.9 eV that responses to the UV band effectively. The facile growth process of the material is an added advantage.

Several groups have attempted to enhance the performance of UV photodetectors by developing heterojunction devices consisting of two different metal oxide semiconductors. For example, Zhao et al. reported the studies of ZnO-Ga_2_O_3_ core-shell heterostructure UV photodetectors, which demonstrated ultra-high responsivity and detectivity due to an avalanche multiplier effect [12, 13]. In this work, a different metal oxide heterojunction, such as NiO/β-Ga_2_O_3_, was investigated to provide a high-performance UV photodetector. Firstly, the lattice mismatch of β-Ga_2_O_3_ and NiO is relatively small. Also, the bandgap of NiO is larger than that of ZnO used in previous study. The p-type behavior of NiO and n-type β-Ga_2_O_3_ has led to several reports on the studies of the electrical properties of NiO/β-Ga_2_O_3_ heterojunction for power electronics applications [14]; however, there is limited report on the use of the heterojunction in photodetector. In this study, the NiO/β-Ga_2_O_3_-based UV photodetector was produced by magnetron sputtering on indium tin oxide (ITO) transparent substrate. The results showed that the NiO/β-Ga_2_O_3_ photodetector exhibited excellent sensitivity to UV light (245 nm) with good stability.

## Methods

Ga_2_O_3_ and NiO ceramic targets (99.99%) were purchased from Zhongnuo Advanced Material (Beijing) Technology Co. Ltd. Sapphire substrate with (0001) plane was purchased from Beijing Physike Technology Co. Ltd. ITO-coated quartz substrate was purchased from Beijing Jinji Aomeng Technology Co. Ltd. All chemical reagents used in the experiments were used without further purification.

β-Ga_2_O_3_ film was prepared by RF magnetron sputtering at room temperature. For characterization, the film was deposited on to sapphire substrate with (0001) plane. Prior to deposition, the substrate was wet-cleaned in a mixed solution of ammonia water, hydrogen peroxide, and deionized water (1:1:3) at 80 °C for 30 min. It was rinsed repeatedly with deionized water and dried using nitrogen to remove surface fouling, which would enhance uniformity and adhesion of the film on the substrate. Sputtering was performed at a pressure of 0.7 Pa with oxygen and argon flowing at a rate of 5 and 95 sccm, respectively. A sputtering power of 200 W was used for a duration of 60 min in the deposition of the film. Finally, the deposited film was annealed in air at 800 °C (60 min) at a heating rate of 10 °C/min.

Crystalline structure of the Ga_2_O_3_ film was studied using X-ray diffraction (XRD, EMPYREAN) and transmission electron microscope (TEM, JEM-2100). Absorption spectra of the Ga_2_O_3_ film on sapphire substrate were measured by UV-Vis spectroscopy (iHR-320), which also provided an estimation on optical bandgap of the film. The surface morphology and thickness of the deposited Ga_2_O_3_ film were characterized using atomic force microscope (AFM, SPA-400) and optical microscope (LEICA DM 2700 M). Elemental analysis of the Ga_2_O_3_ film was performed by X-ray photoelectron spectroscopy (XPS, K-Alpha+). Current-voltage (*J*-*V*) measurement on the NiO/β-Ga_2_O_3_ photodetector was carried out with a Keithley 2400 source meter. All measurements were conducted at room temperature.

## Results and Discussion

**Fig. 1:**
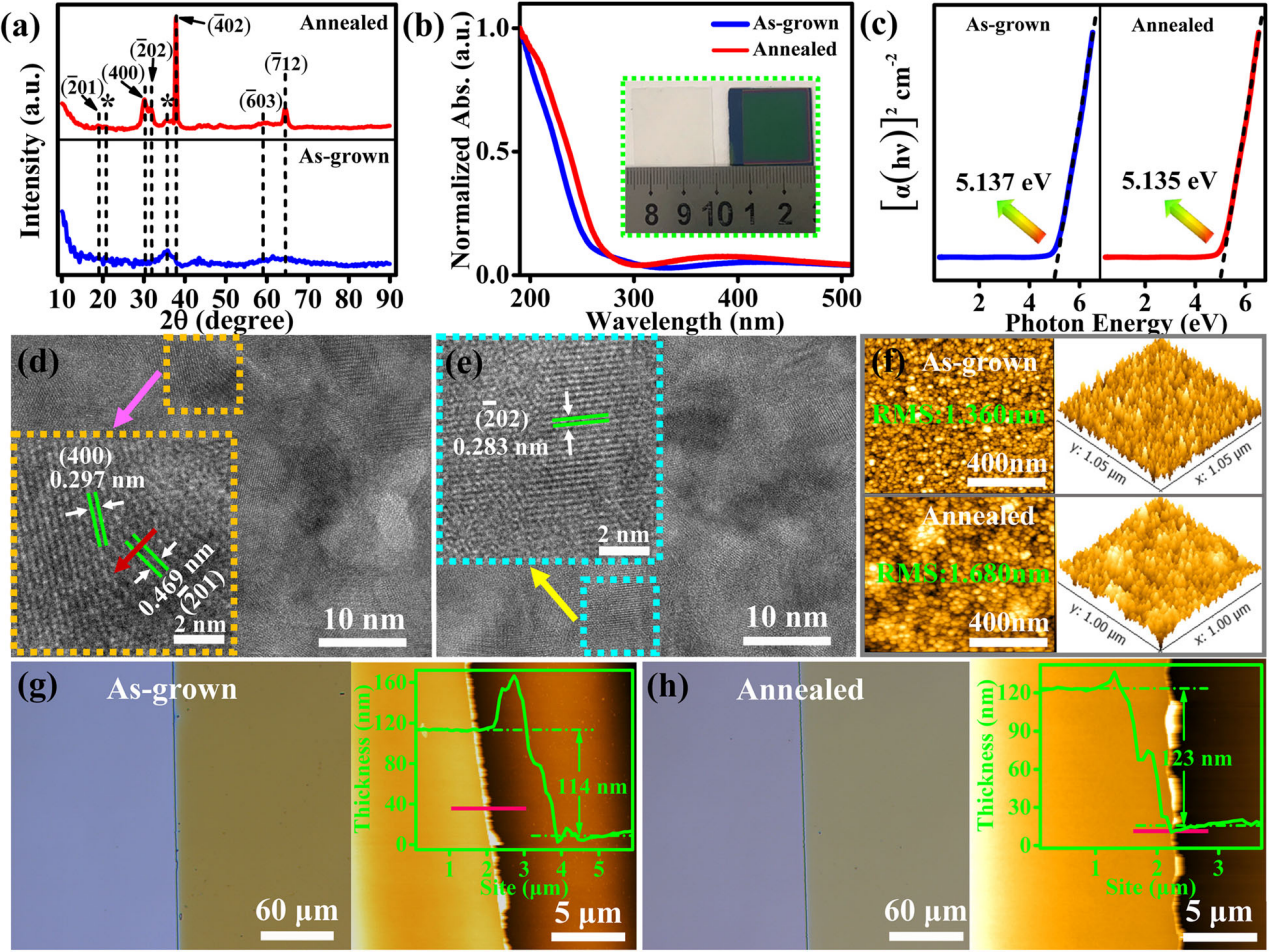
**a** XRD patterns of the β-Ga_2_O_3_ film deposited on sapphire substrate (0001) plane, and the peaks of sapphire substrate are marked as asterisks (*). **b** UV-Vis absorption spectra of the β-Ga_2_O_3_ film. **c** Plots of [α (*hν*)]^2^ versus photon energy. **d–e** TEM and HRTEM images of the β-Ga_2_O_3_ film after annealing. **f** AFM images of the β-Ga_2_O_3_ film. **g–h** Optical and AFM images at the step edge between the film and substrate

Figure 1a show the XRD patterns of the Ga_2_O_3_ film grown on (0001) plane of sapphire substrate before and after annealing. Before annealing, the as-deposited film exhibited an amorphous state as only two peaks (marked as “*”) that associated with the substrate were observed in the pattern. After annealing the film at 800 °C, the XRD pattern showed six characteristic peaks corresponding to crystal planes of β phase of Ga_2_O_3_, which belongs to the monoclinic crystal system. The observed pattern is consistent with previously reported work [15, 16]. These characteristic peaks of the annealed β-Ga_2_O_3_ film revealed good crystallinity with preferential orientation along the family of ($\overline{2}$ 01) crystal planes.

Figure 1d and e are TEM and HRTEM images of the β-Ga_2_O_3_ film after annealing. As shown, the lattice fringe spacing of ($\overline{2}$ 01), (400), and ($\overline{2}$ 02) crystal planes were 4.69 Å, 2.97 Å, and 2.83 Å, respectively, which again suggests good crystallinity and is in good agreement with previously reported work in the literature [17, 18].

AFM image of the β-Ga_2_O_3_ film deposited on sapphire substrate is shown in Fig. 1f. The as-deposited film exhibited a uniform granular surface topography with relatively small root-mean-square (RMS) surface roughness of 1.36 nm. After annealing, the RMS roughness of the film increased to 1.68 nm. Such increase in the RMS roughness after annealing was also reported by Hao et al [19]. It is possible that the annealing treatment could result in surface structural defects. Further studies are required to understand the cause of change in surface morphology after annealing. AFM topography images of the step edge between the film and substrate before and after annealing are shown in Fig. 1 g and h, which the line profiles (in the inset) indicated a film thickness of 114 ± 6.4 nm and 123 ± 2.0 nm (about 8% increase), respectively. The increase in film thickness and RMS after annealing could be that the phase transition from amorphous to crystallinity leads to the nanocrystal grain growth.

UV-Vis absorption spectra of the β-Ga_2_O_3_ films before and after annealing are shown in Fig. 1b. Both films exhibited strong UV absorption in the range of 190–300 nm and almost no absorption in the visible light band. This showed that the annealing treatment did not have a significant effect on the absorption edge. It only resulted in a small red shift of about 10 nm with slight enhancement on the absorption peak. Eq. (1) can be used to estimate the optical bandgap energy (*E*g) of the film.

1 $$ \alpha \left( h\nu \right)=A{\left( h\nu -E\mathrm{g}\right)}^{1/2} $$

where *α* is absorption coefficient, *hν* is photon energy, and *A* is a constant. Taking into account of the film thicknesses measured by AFM, the *E*g of the as-deposited and annealed films can be determined from the plots in Fig. 1c, which indicated a value of 5.137 eV and 5.135 eV, respectively. These values are close to the theoretical *E*g of 4.9 eV for β-Ga_2_O_3_.

**Fig. 2:**
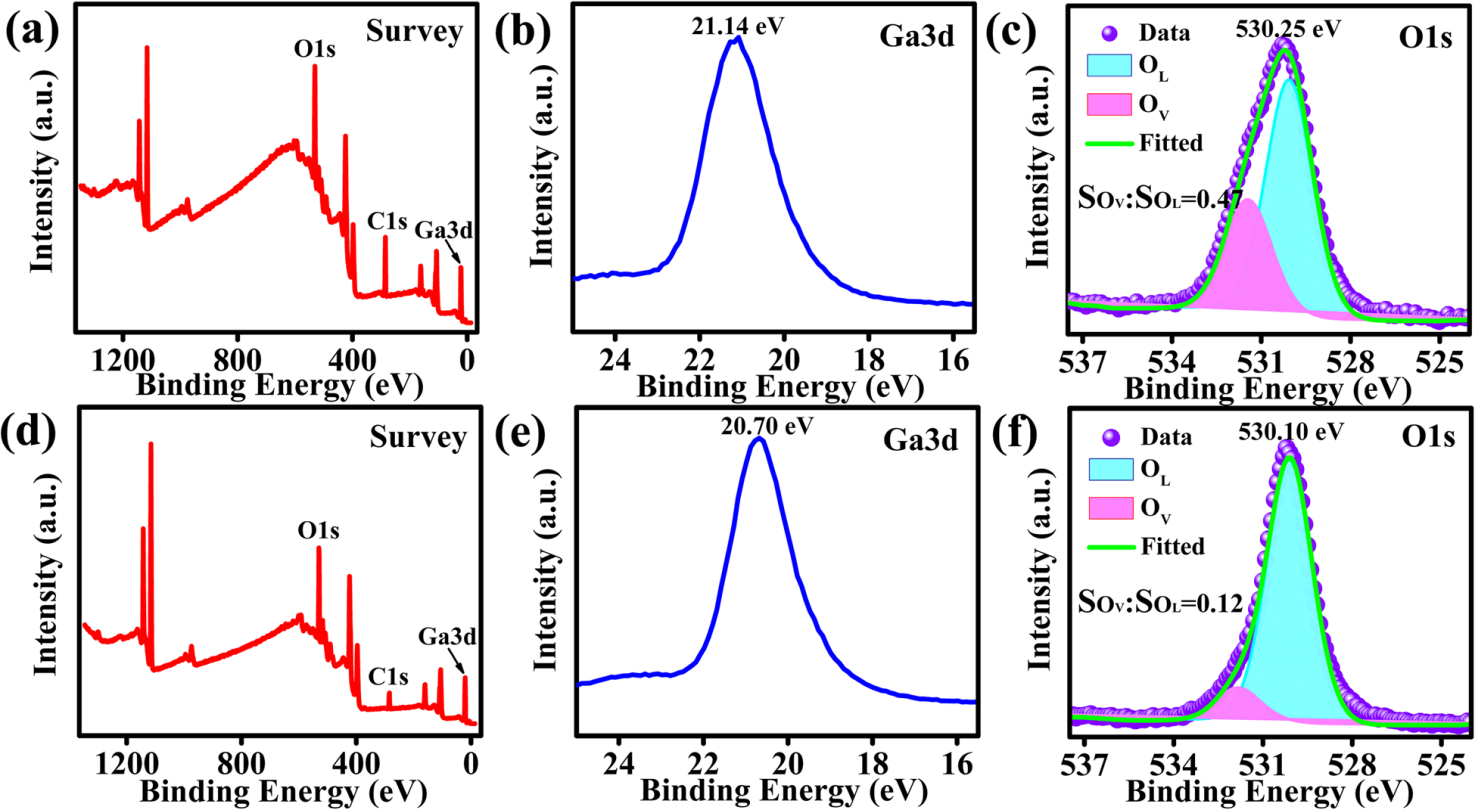
XPS spectra of the β-Ga_2_O_3_ film. Survey scan, Ga 3d, and O1s core level peaks acquired before annealing are shown in **a–c** and after annealing are shown in **d–f**, respectively

XPS spectra of the β-Ga_2_O_3_ film are shown in Fig. 2. Figure 2a–c and d–f show XPS spectra of the full scan, Ga and O elements before and after annealing, respectively. The C element observed from the full scan was adventitious carbon. After annealing, the C 1 s peak was reduced significantly indicating that most carbon was removed during the annealing treatment. The binding energy of Ga3d in Fig. 2 b and e correspond to 21.14 eV and 20.70 eV, respectively, which correspond to the Ga-O bond of the samples, and the binding energy after annealing is reduced by 0.44 eV. The O 1 s peaks were fitted with two components associated with oxygen vacancies (O_V_) and lattice oxygen (O_L_). The area ratios of O_V_ and O_L_ (e.g., S_OV_:S_OL)_ before and after annealing were 0.47 and 0.12, respectively. This suggests an increase in the lattice oxygen atoms due to the annealing treatment leading to crystallization as oxygen atoms move to their appropriate lattice sites.

**Fig. 3:**
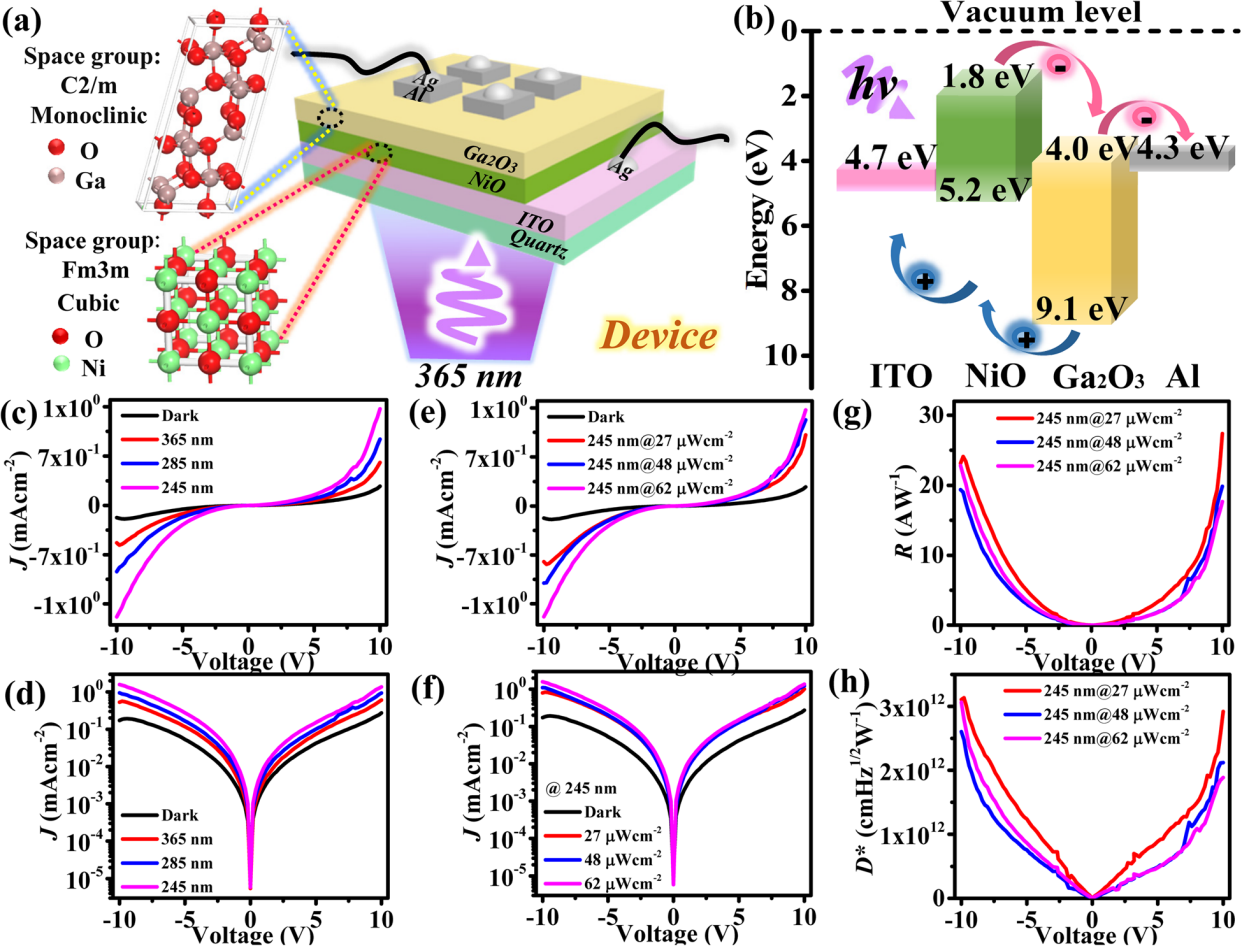
**a** Schematic diagram showing the device structure consisting of ITO/NiO/β-Ga_2_O_3_/Al. **b** Energy band diagram of the photodetector. **c–d** Measured *J-V* and log *J-V* curves, respectively, of the photodetector illuminated with a light of different wavelengths, and under dark conditions. **e–f** Measured *J-V* and log *J-V* curves, respectively, of the photodetector under an UV illumination of 245 nm with different power density. **g–h** Responsivity (*R*) and detectivity (D*), respectively, of the photodetector at different bias voltages under 245 nm light illumination

An UV photodetector consisting of the β-Ga_2_O_3_ film was fabricated. A simple vertical structure was designed for the photodetector, which comprised of ITO/NiO/Ga_2_O_3_/Al. A schematic diagram of the device structure is shown in Fig. 3a. A NiO layer was first sputtered on an ITO-coated quartz substrate after applying the same wet cleaning procedures as the sapphire substrate, and the detailed preparation and characterizations of NiO film were shown in Additional file 1: Figure S1 and Figure S2. Ga_2_O_3_ layer was then sputtered using the above mentioned deposition parameters. The prepared heterojunction was annealed in air at 600 °C for 30 min to avoid heating damage to ITO (with the knowledge that β-Ga_2_O_3_ can be formed at annealing temperature above 550 °C), followed by vapor deposition of Al electrodes (2 × 2 mm^2^) on the surface of Ga_2_O_3_ film. Finally, the Al electrodes and ITO substrate were used as top and bottom electrodes, respectively.

Figure 3b shows the energy band diagram of the photodetector. We calculated the *E*g of NiO film according to Eq. (1) as shown in Additional file 1: Figure S3. The *E*g of NiO film is about 3.4 eV after annealing. The wide bandgap energy of the β-Ga_2_O_3_ (5.1 eV) and NiO (3.4 eV) layers is responsive to UV light. Under UV illumination (*hν*), electrons gain enough energy to transit into the conduction band generating electron-hole pairs. These photogenerated electron-hole pairs were separated by the built-in electric field and collected by the respective electrodes. Here, the heterostructure with appropriate band alignment can facilitate the charge separation and collection.

The performance of the heterojunction photodetector was studied from the measured *J*-*V* and log *J*-*V* plots, which were acquired from the backlighting incident device. Figure 3 c and d illustrate the *J*-*V* and log *J*-*V* curves of the photodetector illuminated with different wavelength lights and under dark condition, respectively. When the photodetector was illuminated by a 245-nm UV light at 27 μWcm^−2^, a drastic increase of a current density, up to 1.38 mAcm^−2^, was observed at an applied voltage of 10 V. The current density also increases when illuminated with 285 and 365 nm UV lights. However, more electron-hole pairs can be effectively excited by 245 nm UV light compared with other two UV lights, showing the deep UV detection of the device.

*J*-*V* and log *J*-*V* curves of the photodetector were measured under an UV illumination of 245 nm with varying power density, as shown in Fig. 3 e and f, respectively. Measurements were performed under dark and UV light conditions. The current density increases with the light intensity under a 245-nm UV illumination which suggests that the photodetector has the ability to generate photocurrent in response to 245 nm UV light.

The effect of bias voltage on the responsivity (*R*) of the device is shown in Fig. 3g. *R* is related to the photocurrent density (*J*_ph_) according to Eq. (2) [5]:

2 $$ R={J}_{\mathrm{ph}}/{P}_{\mathrm{opt}} $$

where *P*_opt_ is photon power density having a value of 1.5 mWcm^−2^. An increase in *R* was evident from Fig. 3g as the bias voltage of the device increases under fixed photon power density. The maximum *R* was 27.43 AW^−1^ measured under a 245-nm illumination (27 μWcm^−2^) at the bias voltage of 10 V.

Detectivity (D*) is another important parameter for evaluating the performance of photodetectors. D* of the photodetector can be calculated using Eq. (3) as follows [20, 21]:

3 $$ {D}^{\ast }=R/{\left(2q\left|{J}_{\mathrm{d}}\right|\right)}^{1/2} $$

where *q* is absolute electron charge (1.602 × 10^−19^ C) and *J*_d_ is dark current density. The relationship between D* and the bias voltage is shown in Fig. 3h, which shows an increase in D* as the bias voltage increases. The maximum D* was 3.14 × 10^12^ cmHz^1/2^ W^−1^ measured under a 245-nm illumination (27 μWcm^−2^) at the bias voltage of − 10 V. Based on the values of *R* and D*, the NiO/β-Ga_2_O_3_ photodetector demonstrated high performance in UV detection, compared with other NiO-based and Ga_2_O_3_-based UV detectors shown in Table 1.

**Table 1:** Comparison of characteristic parameters of other NiO-based and Ga_2_O_3_-based UV detectors

Device	Preparation method	Wavelength	Bias voltage	*R* (AW^−1^)	Year	Ref.
ITO/NiO/β-Ga_2_O_3_/Al	RF magnetron sputtering	245 nm	10 V	27.43	–	This work
Ni/NiO/ZnO/FTO	RF/DC sputtering	400 nm	− 5 V	3.85	2015	[22]
Al/ZnO/NiO/ITO	Sol-gel/spin-coating	350 nm	− 1 V	10.2	2014	[23]
ZnO/Ga_2_O_3_ microwires	CVD	251 nm	–	9.7 × 10^−3^	2017	[12]
Graphene/β-Ga_2_O_3_	CVD	–	20 V	39.3	2016	[11]
GaN/Sn:Ga_2_O_3_	PLD	254 nm	–	3.05	2018	[24]
Graphene/β-Ga_2_O_3_	Mechanical exfoliation	254 nm	–	29.8	2018	[25]
β-Ga_2_O_3_/Nb:SrTiO_3_	RF magnetron sputtering	254 nm	− 10 V	43.31	2017	[26]

## Conclusions

In conclusion, β-Ga_2_O_3_ film was prepared by RF magnetron sputtering and exhibited good crystallinity after annealing at 800 °C. The wide bandgap material revealed strong UV absorption in the range of 190–300 nm. The deep UV photodetector based on NiO/β-Ga_2_O_3_ heterostructure was highly sensitive to 245 nm UV light with high responsivity (*R*) and detectivity (D*) of up to 27.43 AW^−1^ and 3.14 × 10^12^ cmHz^1/2^ W^−1^, respectively. It is believed that the performances of the UV photodetector can be further improved by means of doping or optimizing the device structure.

## Supplementary information

**Additional file 1: Figure S1.** XRD patterns of the NiO film deposited on quartz substrate before and after annealing (blue line and red line). **Figure S2.** TEM and HRTEM images of the NiO film after annealing. **Figure S3.** (a) UV-Vis absorption spectra of the NiO film before and after annealing (blue line and red line), the inset is the picture of the prepared film. (b) Plots of [α (*hν*)]^2^ versus photon energy. **Figure S4.** AFM images of the NiO film after annealing. **Figure S5.** The ohmic contact of Al-*β*-Ga_2_O_3_-Al.

## Data Availability

The conclusions made in this manuscript are based on the data (main text and figures) presented and shown in this paper.

## Abbreviations

AFM: Atomic force microscope
ITO: Indium tin oxide
TEM: Transmission electron microscope
UV: Ultraviolet
XPS: X-ray photoelectron spectroscopy
XRD: X-ray diffraction
