# Supplementary material for: High-Performance Deep Ultraviolet Photodetector Based on NiO/β-Ga2O3 Heterojunction
Source: Nanoscale Res Lett. 2020 Feb 22;15:47. doi: 10.1186/s11671-020-3271-9 (PMC7036083; doi:10.1186/s11671-020-3271-9)
Supplement: Supplementary file 1 — Additional file 1: Figure S1. XRD patterns of the NiO film deposited on quartz substrate before and after annealing (blue line and red line). Figure S2. TEM and HRTEM images of the NiO film after annealing. Figure S3. (a) UV-Vis absorption spectra of the NiO film before and after annealing (blue line and red line), the inset is the picture of the prepared film. (b) Plots of [α (hν)]2 versus photon energy. Figure S4. AFM images of the NiO film after annealing. Figure S5. The ohmic contact of Al-β-Ga2O3-Al. [file 11671_2020_3271_MOESM1_ESM.docx]

*Additional file* ***1*** *for*

High-performance deep ultraviolet photodetector based on

NiO/β-Ga_2_O_3_ heterojunction

Menghan Jia^1,2,3^, Fang Wang^1,2,3^, Libin Tang^2,3^*, Jinzhong Xiang^4^*, Kar Seng Teng^5^*, Shu Ping Lau^6^

^1^ School of Materials Science and Engineering, Yunnan University, Kunming 650091, China

^2^ Kunming Institute of Physics, Kunming 650223, China

^3^ Yunnan Key Laboratory of Advanced Photoelectric Materials & Devices, Kunming 650223, China

^4^ School of Physics and Astronomy, Yunnan University, Kunming 650091, China

^5^ College of Engineering, Swansea University, Bay Campus, Fabian Way, Swansea SA1 8EN, United Kingdom

^6^ Department of Applied Physics, The Hong Kong Polytechnic University, Hung Hom, Kowloon, Hong Kong, China

1. **The preparation of NiO film**

NiO film was prepared by RF magnetron sputtering at room temperature. For characterization, the film was deposited on quartz substrate. Prior to deposition, the substrate was wet-cleaned in a mixed solution of ammonia water, hydrogen peroxide and deionized water (1:1:3) at 80 °C for 30 min. It was rinsed repeatedly with deionized water and dried using nitrogen to remove surface fouling, which would enhance uniformity and adhesion of the film on the substrate. Sputtering was performed at a pressure of 0.6 Pa with oxygen and argon flowing at a rate of 35 and 35 sccm, respectively. A sputtering power of 200 W was used for a duration of 60 min in the deposition of the film. Then, the deposited film was annealed in air at 600 °C at a heating rate of 10 °C/min.

1. **XRD patterns of the NiO film**

Figure S1 shows the XRD patterns of the NiO film grown on quartz substrate before and after annealing. Before annealing, the diffraction peak is not sharp, which indicates that the crystallization of the film still needs to be improved. After annealing, the XRD pattern showed three characteristic peaks corresponding to (111), (200) and (220) crystal planes of cubic phase of NiO, respectively. A peak (marked as “*”) associated with the substrate was also observed in the pattern. The stronger diffraction peaks revealed the better crystallinity after annealing.

**
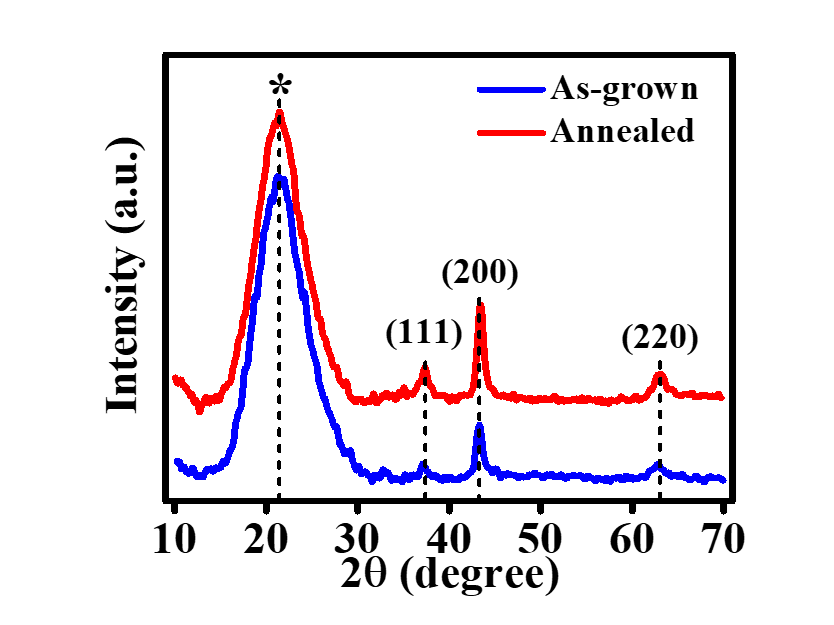
**

**Fig. S1.** XRD patterns of the NiO film deposited on quartz substrate before and after annealing (blue line and red line).

1. **TEM images of the NiO film**

As shown in Fig. S2, the NiO film exhibits good crystallinity. The lattice fringe spacing of (200) and (111) crystal planes were 2.08 Å and 2.42 Å, respectively, which is consistent with the XRD results in Fig. S1.

**
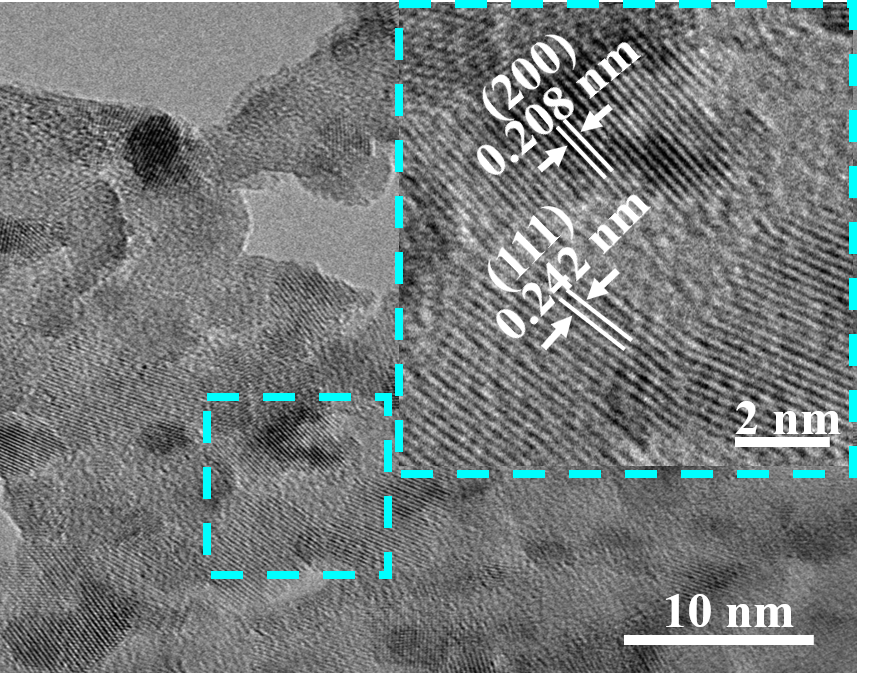
**

**Fig. S2.** TEM and HRTEM images of the NiO film after annealing.

1. **Optical characteristics of the NiO film**

UV-Vis absorption spectra of the NiO film before and after annealing are shown in Fig. S3(a). Both films exhibited strong UV absorption in the range of 190-370 nm, the annealed film has a weaker absorption in the visible light band. It can also be found that annealing makes the absorption edge steeper. The optical bandgap energy (*E*g) of the as-grown and annealed films can be determined from the plots in Fig. S3(a), which indicated a value of 2.9 and 3.4 eV, respectively. The value of annealed film is close to the theoretical *E*g of 3.6 eV. Optical band gap of NiO film can be calculated using the following formula:

${\alpha\left( h\nu\right)=A\left( h\nu-Eg \right)}^{1/2}$ (1)

where *α* is absorption coefficient, *hν* is photon energy and A is a constant.


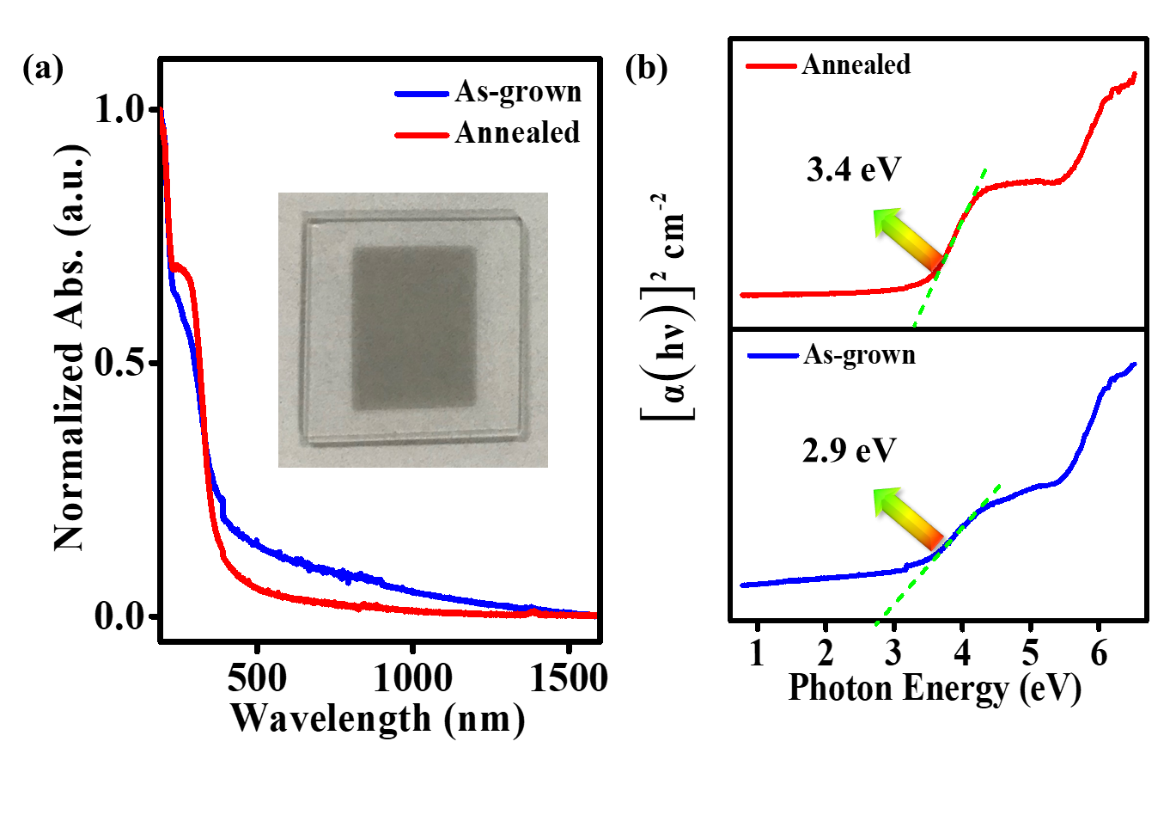


**Fig. S3.** (a) UV-Vis absorption spectra of the NiO film before and after annealing (blue line and red line), the inset is the picture of the prepared film. (b) Plots of [α(*hν*)]^2^ versus photon energy.

1. **AFM images of the NiO film**

As shown in Fig. S4(a), the annealed NiO film exhibited an uniform granular surface topography with relatively small root-mean-square (RMS) surface roughness of 1.37 nm. AFM topography image of the step edge between the film and quartz substrate is shown in Fig. S4(b), the line profile (in the inset) indicated a film thickness of 131 ± 3.4 nm.


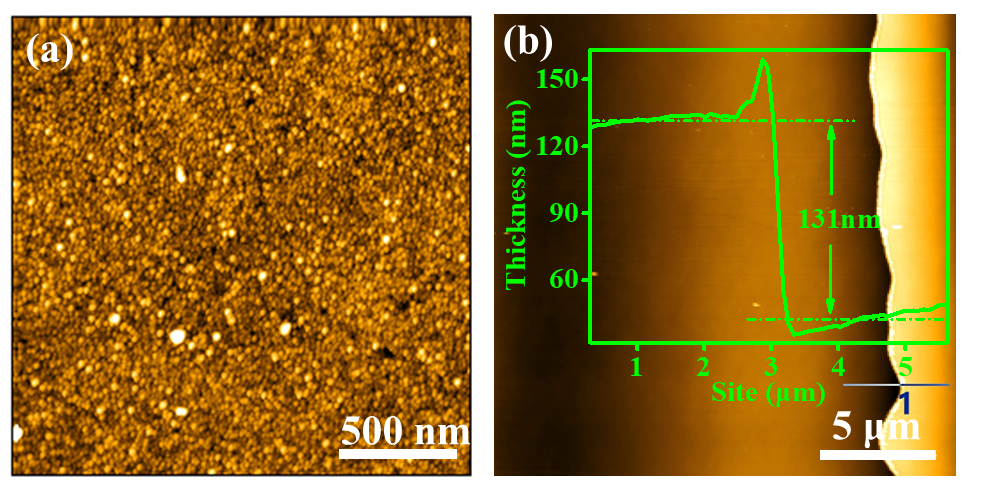


**Fig. S4.** AFM images of the NiO film after annealing.

1. **Ohmic contact**

The ohmic contact between material and electrode has been measured, the result is shown in Fig. S5, it can be seen that the contact between material and electrode is the ohmic contact.


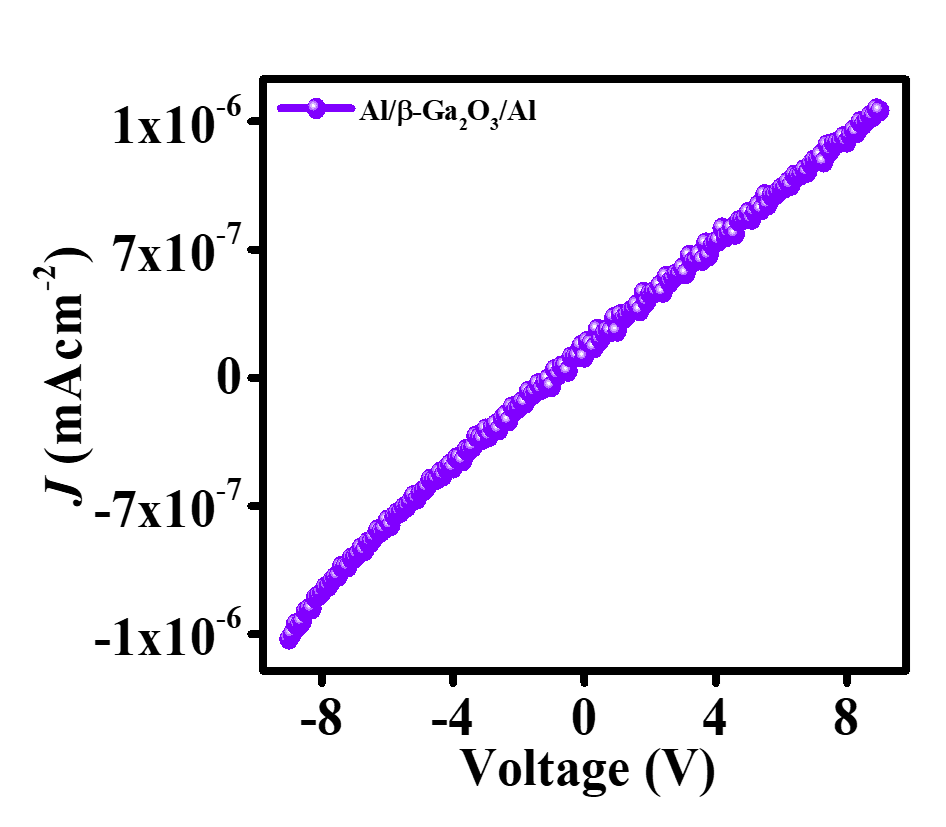


**Fig. S5.** The ohmic contact of Al-β-Ga_2_O_3_-Al.
